# Additive effects of postchallenge hyperglycemia and low-density lipoprotein particles on the risk of arterial stiffness in healthy adults

**DOI:** 10.1186/1476-511X-13-179

**Published:** 2014-11-27

**Authors:** Cheng Ding, Sandy Huey-Jen Hsu, Yong-Jian Wu, Ta-Chen Su

**Affiliations:** State Key Laboratory of Cardiovascular Disease, Department of Cardiology, Cardiovascular Institute, Fuwai Hospital and National Center for Cardiovascular Diseases, Chinese Academy of Medical Sciences & Peking Union Medical College, Beijing, 100037 China; Department of Laboratory Medicines, National Taiwan University Hospital, Taipei, Taiwan; Departments of Internal Medicine and Cardiovascular Center, National Taiwan University Hospital, 7, Chung-Shan South Road, Taipei, 100 10002 Taiwan

**Keywords:** Postchallenge hyperglycemia, sdLDL-C, LDL-C, Arterial stiffness, OGTT

## Abstract

**Background:**

To determine the effects of post-challenge hyperglycemia potentiate low-density lipoprotein cholesterol (LDL) particles on the risk of arterial stiffness in non-diabetic adults.

**Methods:**

During 2009–2011, 592 adults without clinical diabetes (fasting glucose <7.0 mmol/L) or known coronary heart disease or stroke were recruited. All subjects underwent standard 75-g oral glucose tolerance test (OGTT) after overnight fasting. The glucose area under curve (GluAUC) after OGTT was defined as the postchallenge glucose load. Levels of LDL-C and small dense LDL-C (sdLDL-C) were measured. Arterial stiffness in terms of brachial–ankle pulse wave velocity (baPWV) was also measured.

**Results:**

The baPWV in tertile distributions were significantly associated with all conventional cardiovascular risk factors, LDL-C, and sdLDL-C. Multivariate logistic regression analyses revealed that LDL-C (or sdLDL-C) combined with one of the seven glycemic indices (glucose levels at 0, 30, 60, 90, and 120 min; GluAUC; HbA1C) was associated with arterial stiffness after covariates being adjusted. Further interaction analyses showed only concurrent higher levels of both glycemic indices and atherogenic LDL-C or sdLDL-C have significant risk for arterial stiffness.

**Conclusions:**

Additive effects of both postchallenge hyperglycemia and LDL subclass particles potentiate the risk of arterial stiffness. The adverse joint effects of hyperlipidemia and postchallenge hyperglycemia on subclinical cardiovascular function provide important information in primary prevention of cardiovascular disease in subjects without clinical diabetes.

## Background

Several epidemiological and pathological studies have shown that diabetes is an independent risk factor for cardiovascular disease (CVD) in men and women [1]. As the recommendation from the 2002 National Cholesterol Education Program Adult Treatment Panel III, diabetes mellitus has been considered as a coronary heart disease (CHD) equivalent [2], although residual controversy has continued into this decade. Recently, it was demonstrated that some diabetic CHD patients may be at a greater risk of CVD morbidity and mortality compared with nondiabetic CHD patients [3]. Moreover, the Heart Protection Study provides direct evidence that cholesterol-lowering therapy is beneficial for people with diabetes even if they have not developed CHD or display elevated cholesterol concentrations [4]. Another study also showed that cholesterol-lowering therapy is efficacious in reducing the risk of first CVD events in patients with type 2 diabetes without high low-density lipoprotein (LDL) cholesterol (LDL-C) levels [5]; however, the interaction between glucose and the cholesterol concentrations remains unclear. On the other hand, about one-third of American adults and two-thirds of CHD patients have abnormal glucose metabolism [6, 7]. Most of these high-risk individuals are still within nondiabetic glucose levels (fasting plasma glucose, FPG < 7.0 mmol/l or 2-h plasma glucose <11.1 mmol/l), but show prediabetes classification with impaired glucose tolerance (FPG <7.0 mmol/l and 2-h plasma glucose ≥7.8 and <11.1 mmol/l) or diabetes classification after an oral glucose tolerance test (2-h plasma glucose >11.1 mmol/l) or an ingestion of a meal. Several studies have shown that postchallenge glucose levels are more strongly associated with CVD compared with fasting glucose levels [8, 9], besides, glucose levels after a glucose challenge exhibit continuous linear relationships with both CVD death and all-cause mortality risk [10]. Alternatively, the importance of 2-h plasma glucose levels during oral glucose tolerance test (OGTT) is well established, but the importance of postchallenge hyperglycemia remains unclear [11].

Although the above studies indicated the importance of postprandial glucose levels, fasting plasma glucose and glycated hemoglobin (HbA1c) levels still play important roles in the assessment of glycemic levels, whereas postchallenge glucose values are often neglected [12]. This may be because of the inconvenience and cost of measuring these parameters in daily clinical practice [13]. The most common clinical phenotypes of dyslipidemia in individuals with impaired glucose metabolism include low high-density lipoprotein cholesterol (HDL-C) levels, hypertriglyceridemia, and a higher ratio of small dense LDL-cholesterol (sdLDL-C) [14–17]. Higher levels of sdLDL particles are considered to be more atherogenic compared with large buoyant LDL-C particles; therefore, sdLDL-C is considered to be an important and independent predictor of CVD [18, 19].

Brachial-ankle pulse wave velocity (baPWV) can be noninvasively measured simply by wrapping the four extremities with blood pressure cuffs, and it serves as a simple marker of vascular damage and cardiovascular risk [20, 21]. Earlier studies on experimental atherosclerosis in monkeys demonstrated that the aortic pulse wave velocity (PWV) increased with the development of atherosclerosis [22]. The Rotterdam study, a population-based study including more than 3,000 subjects, demonstrated a strong association of aortic PWV with intima-media thickness (IMT) and the severity of plaques as measured by ultrasonographic tests [23]. In addition, from our previously work, which have demonstrated that postchallenge hyperglycemic spikes correlate with arterial stiffness which was independent of confounding factors in adults without diabetes [24].

Thus, we propose that measuring sdLDL particle concentrations in a cohort study involving individuals without clinical diabetes would be helpful in evaluating its earlier role of dyslipidemia-glucose hypothesis in atherosclerosis; we conducted this study among healthy adults without diabetes with the aim of investigating whether postchallenge hyperglycemia and LDL subclass particles exerted additive effects on arterial stiffness.

## Results

The cardiovascular characteristics of all participants were compared according to the tertile distribution of sdLDL levels; subjects without previously known clinical diabetes that displayed 2-h glucose ≥11.1 mmol/l after OGTT were considered OGTT diabetes (Table 1). A strong correlation between different glycemic indices such as OGTT diabetes, GluAUC and fasting glucose across sdLDL-C tertile (Table 1) was noted. The conventional risk factors for CVD and diabetes, except smoking and alcohol consumption, showed statistically significant differences in the trend test across the three groups. Subjects with higher sdLDL levels exhibited more conventional risk factor for CVD and diabetes. Higher LDL-C and sdLDL/LDL ratios were also noted across the higher sdLDL levels. Most conventional risk factors and the novel risk factors of sdLDL and GluAUC were significantly associated with baPWV across the tertile distribution (Table 2). The upper tertile of baPWV subjects associated with significantly higher glucose profiles including OGTT diabetes, FPG, GluAUC and HbA1C and lipid profiles such as cholesterol, triglyceride, LDL-C, sdLDL-C and sdLDL-C/LDL-C ratio. In contrast, levels of large LDL-C were borderline but significantly associated with baPWV. Additionally, there are no significant associations between baPWV and statin-users, and between baPWV and renal function index of estimated glomerular filtration rate.

**Table 1:** **Cardiovascular characteristics of participants according to tertile distribution of small dense-low-density lipoprotein cholesterol (sdLDL-C)**

	sdLDL-C, mmol/L			
	Upper	Middle	Lower	***p*** -value
	≥ 37.7	24.0-37.7	< 24.0	
Characteristics	N = 197	N = 197	N = 198	
Age, years	45.82 ± 7.61	44.41 ± 7.96	45.13 ± 7.99	0.390
Male, %	91.88	90.16	78.35	<.001
BMI, kg/m^2^	25.79 ± 2.95	25.26 ± 3.62	24.19 ± 3.19	<.001
Waist, cm	87.28 ± 7.94	85.61 ± 10.1	81.87 ± 9.04	<.001
Smoking, %	20.81	14.95	14.29	0.083
Alcohol, %	23.86	21.65	14.8	0.025
Hypertension, %	27.92	18.04	14.8	0.001
Systolic BP, mmHg	126.04 ± 14.67	121.23 ± 10.91	117.44 ± 13.23	<.001
Diastolic BP, mmHg	77.53 ± 9.79	74.18 ± 8.19	71.28 ± 9.51	<.001
OGTT diabetes, %	9.14	4.12	3.57	0.017
Fasting glucose, mmol/L	5.45 ± 1.21	5.15 ± 0.78	5.03 ± 0.93	<.001
GluAUC, mmol/L	32.53 ± 10.07	29.24 ± 7.01	26.64 ± 8.13	<.001
HbA1C, %	5.85 ± 1.05	5.55 ± 0.46	5.56 ± 0.69	<.001
Cholesterol, mmol/L	5.98 ± 0.88	5.31 ± 0.62	4.7 ± 0.65	<.001
Triglyceride, mmol/L	2.39 ± 1.49	1.48 ± 0.75	0.99 ± 0.41	<.001
HDL-C, mmol/L	1.22 ± 0.3	1.27 ± 0.31	1.37 ± 0.3	<.001
LDL-C, mmol/L	3.55 ± 0.89	3.15 ± 0.61	2.59 ± 0.52	<.001
sdLDL-C/LDL-C	0.39 ± 0.13	0.26 ± 0.07	0.18 ± 0.04	<.001

OGTT Diabetes: subjects’ results with OGTT two hour value >11.1 mmol/l.

*Abbreviations*: *OGTT* oral glucose tolerance test, *GluAUC* glucose area under curve after OGTT.

**Table 2:** **Cardiovascular characteristics of participants according to tertile distribution of brachial–ankle pulse wave velocity (baPWV)**

		baPWV		
	Upper	Middle	Lower	***p*** -value
	≥ 1438.3	1322.5-1438.3	< 1322.5	
Characteristics	N = 198	N = 197	N = 197	
Age, years	48.18 ± 7.35	45.12 ± 7.53	42.24 ± 7.58	<.001
Male, %	92.93	87.24	81.03	0.004
BMI, kg/m^2^	25.43 ± 3.23	25.27 ± 3.26	24.58 ± 3.48	0.010
Waist, cm	86.34 ± 8.48	85.23 ± 9.06	83.37 ± 10.17	0.001
Smoking, %	20.2	13.71	16.75	0.359
Alcohol, %	22.22	21.83	15.23	0.081
Hypertension, %	33.84	17.77	8.12	<.001
Systolic BP, mmHg	129.61 ± 14.58	121.52 ± 10.71	113.59 ± 9.25	<.001
Diastolic BP, mmHg	79.50 ± 9.71	75.02 ± 7.85	68.59 ± 7.44	<.001
OGTT diabetes, %	9.6	4.06	1.52	<.001
Fasting glucose, mmol/L	5.39 ± 0.92	5.18 ± 0.89	4.99 ± 0.86	<.001
GluAUC, mmol/L	32.38 ± 9.84	29.09 ± 7.82	27.37 ± 7.97	<.001
HbA1C, %	5.77 ± 0.87	5.62 ± 0.57	5.51 ± 0.72	<.001
Cholesterol, mmol/L	5.50 ± 0.96	5.37 ± 0.93	5.14 ± 0.74	<.001
Triglyceride, mmol/L	1.85 ± 1.43	1.61 ± 0.94	1.41 ± 0.97	<.001
HDL-C, mmol/L	1.24 ± 0.27	1.30 ± 0.32	1.33 ± 0.33	0.005
LDL-C, mmol/L	3.28 ± 0.87	3.12 ± 0.76	2.90 ± 0.71	<.001
sdLDL, mmol/L	0.96 ± 0.47	0.86 ± 0.41	0.73 ± 0.33	<.001
Large LDL-C, mmol/L	2.31 ± 0.71	2.28 ± 0.64	2.18 ± 0.60	0.037
sdLDL-C/LDL-C	0.30 ± 0.14	0.27 ± 0.12	0.25 ± 0.11	<.001
eGFR, ml/min	87.26 ± 19.60	88.91 ± 20.49	89.39 ± 20.05	0.323
Statin-users, %	13.13	8.63	9.64	0.257

OGTT Diabetes: subjects’ results with OGTT two hour value >11.1 mmol/l.

*Abbreviations*: *OGTT* oral glucose tolerance test, *GluAUC* glucose area under curve after OGTT, *sdLDL-C* small dense LDL-C, Large LDL-C = LDL-C – sdLDL-C, *eGFR* estimated glomerular filtration rate.

Multivariate logistic regression models were applied to elucidate the impact of different glycemic indices, including post-OGTT glucose levels, GluAUC, and HbA1C, combined with LDL-C (Table 3) or with sdLDL-C (Table 4) on the risk of baPWV. The results demonstrated that fasting glucose levels were not associated with arterial stiffness after controlling for other covariates; however, postchallenge glucose levels at 30, 60, 90, and 120 min and GluAUC were significantly and positively associated with arterial stiffness. Furthermore, the joint effects of postchallenge glucose levels and GluAUC with either LDL-C or sdLDL-C were significantly linked to arterial stiffness. On the other hand, when the joint effects of both LDL-C and fasting glucose levels or LDL-C and HbA1C were considered, the two glycemic indices no longer had a significant influence on increased baPWV (Table 3). Similar findings were observed when LDL-C was replaced with sdLDL-C in the same multivariate models (Table 4). Fasting glucose and HbA1C did not significantly influence the risk of arterial stiffness in the initial multivariate analyses (Tables 3 and 4).

**Table 3:** **Multiple logistic regression analyses for the risk [odds ratios (95% confidence intervals)] of higher brachial–ankle pulse wave velocity (baPWV ≥75** ^**th**^ **percentile) focused on the combined effects of LDL-C and different glycemic indices**

	Basic models ^a^	Model 1	Model 2	Model 3	Model 4	Model 5	Model 6	Model 7
**LDL-C**	1.62(1.27-2.06)^‡^	1.59(1.24-2.02)^‡^	1.55(1.21-1.99)^‡^	1.53(1.19-1.95)^‡^	1.56(1.22-1.99)^‡^	1.57(1.23-2.00)^‡^	1.54(1.20-1.97)^‡^	1.60(1.25-2.04)^‡^
Glucose,								
0 min	1.24(0.99-1.55)*	1.19(0.96-1.48)	—	—	—	—	—	—
30 min	1.23(1.10-1.38)*	—	1.21(1.08-1.35)^†^	—	—	—	—	—
60 min	1.15(1.07-1.23)*	—	—	1.13(1.05-1.22)*	—	—	—	—
90 min	1.12(1.05-1.20)*	—	—	—	1.10(1.03-1.18)*	—	—	—
120 min	1.12(1.04-1.20)*	—	—	—	—	1.10(1.02-1.19)*	—	—
GluAUC	1.09(1.04-1.14)*	—	—	—	—	—	1.08(1.03-1.13)*	—
HbA1C	1.34(1.02-1.76)*	—	—	—	—	—	—	1.30(0.99-1.72)

^a^Basic models included LDL-C or one of the seven glycemic indices after controlling for age, male gender, BMI, hypertension, smoking, and alcohol consumption habit.

p-value: *<0.05, ^†^<0.01, ^‡^<0.001.

Models 1 to 7 combined both LDL-C and one of the seven glycemic indices after controlling for age, male gender, BMI, hypertension, smoking, and alcohol consumption.

**Table 4:** **Multiple logistic regression analyses for the risk [odds ratios (95% confidence intervals)] of higher brachial–ankle pulse wave velocity (baPWV ≥75** ^**th**^ **percentile) focused on the combined effects of sdLDL-C and different glycemic indices**

	Basic models ^a^	Model 1	Model 2	Model 3	Model 4	Model 5	Model 6	Model 7
**sdLDL-C**	2.51(1.56-4.05)^‡^	2.38(1.46-3.87)^‡^	2.17(1.32-3.57)^‡^	2.12(1.29-3.48)^‡^	2.22(1.36-3.64)^‡^	2.30(1.42-3.75)^‡^	2.16(1.31-3.54)^‡^	2.37(1.46-3.86)^‡^
Glucose,								
0 min	1.24(0.99-1.54)	1.15(0.93-1.43)	—	—	—	—	—	—
30 min	1.23(1.10-1.38)^†^	—	1.18(1.05-1.33)^†^	—	—	—	—	—
60 min	1.15(1.07-1.23)*	—	—	1.12(1.04-1.20)*	—	—	—	—
90 min	1.12(1.04-1.20)*	—	—	—	1.09(1.02-1.17)*	—	—	—
120 min	1.11(1.03-1.20)*	—	—	—	—	1.09(1.01-1.18)*	—	—
GluAUC	1.09(1.04-1.14)*	—	—	—	—	—	1.07(1.02-1.12)*	—
HbA1C	1.36(1.02-1.75)*	—	—	—	—	—	—	1.22(0.93-1.60)

^a^Basic models included sdLDL-C or one of the seven glycemic indices after controlling for age, male gender, BMI, hypertension, smoking, and alcohol consumption habit.

p-value: *<0.05, ^†^<0.01, ^‡^<0.001.

Models 1 to 7 combined both sdLDL-C and one of the seven glycemic indices after controlling for age, male gender, BMI, hypertension, smoking, and alcohol consumption.

However, further multivariate analyses using interaction analyses between glycemic indices and atherogenic lipoprotein particles revealed that that only group of concurrent with highest tertile levels of LDL-C (or sdLDL-C) and higher half levels glycemic indices have significant risk for arterial stiffness and this is independent of fasting status (either fasting or postchallenge) or HbA1C levels (Table 5).

**Table 5:** **Multivariate logistic regression analyses for the interaction between atherogenic lipids and glycemic indices (GI) on the risk of arterial stiffness (baPWV ≥75** ^**th**^ **percentile)**

	AC	Glucose 30	Glucose 60	Glucose 90	Glucose 120	Glucose AUC	HbA1C
**LDL-C ≥2/3 & GI ≥50%**	2.63(1.59,4.35)^‡^	2.13(1.29,3.52)^†^	2.26(1.36,3.75)^†^	1.85(1.12,3.06)^†^	2.21(1.34,3.65)^†^	2.27(1.38,3.74)^†^	2.50(1.49,4.19)^‡^
**LDL-C ≥2/3 & GI <50%**	1.69(0.99,2.89)	1.28(0.74,2.21)	1.15(0.67,1.97)	1.55(0.90,2.65)	1.78(1.04,3.04)*	1.40(0.81,2.41)	1.89(1.13,3.16)*
**LDL-C <2/3 & GI ≥50%**	1.16(0.75,1.80)	0.68(0.43,1.08)	0.65(0.41,1.02)	0.71(0.45,1.12)	0.98(0.63,1.54)	0.84(0.53,1.32)	1.18(0.76,1.85)
**LDL-C <2/3 & GI <50%**	1	1	1	1	1	1	1
**sdLDL-C ≥2/3 & GI ≥50%**	2.05(1.26,3.33)^†^	1.70(1.02,2.82)*	1.63(1.00,2.66)*	1.47(0.91,2.40)	1.74(1.06,2.84)*	1.84(1.12,3.02)^†^	1.97(1.18,3.29)^†^
**sdLDL-C ≥2/3 & GI <50%**	1.20(0.68,2.13)	0.79(0.51,1.23)	1.34(0.76,2.35)	1.25(0.70,2.21)	1.38(0.79,2.41)	1.10(0.62,1.94)	1.50(0.89,2.54)
**sdLDL-C <2/3 & GI ≥50%**	1.12(0.72,1.73)	1.11(0.64,1.93)	0.87(0.56,1.36)	0.71(0.46,1.12)	0.99(0.64,1.54)	0.88(0.56,1.38)	1.21(0.78,1.88)
**sdLDL-C <2/3 & GI <50%**	1	1	1	1	1	1	1

p-value: *<0.05, ^†^<0.01, ^‡^<0.001.

All models were after adjusting for age, male gender, BMI, hypertension, alcohol drinking and current smoker, in addition to glycemic indices and LDL-C (or sdLDL-C).

Levels of LDL-C (130 mg/dL) and sdLDL-C (37 mg/dL) were set at cut point of ≥ upper tertile (2/3) (66.7^th^ percentile) in the models.

## Discussion

To the best of our knowledge, the present study is the first to demonstrate the concurrent presence of both atherogenic lipoprotein particles (LDL and sdLDL) and postchallenge hyperglycemia as risk factors of arterial stiffness in nondiabetic adults. The novel finding of significant interactions between LDL subclass particles and different glycemic indices also indicate an additive effect between these two risk factors on arterial stiffness. In addition to postchallenge glucose levels, both LDL-C and sdLDL-C levels were significantly related to an increased risk of arterial stiffness. Another novel result of our study found that sdLDL-C might play an important role as LDL-C as it being closely related to the early atherosclerosis maker of baPWV.

Formerly studies have demonstrated that postchallenge hyperglycemia status predisposes a higher risk of atherosclerosis or CVD that can be attributed to the rapid increase in oxidative stress after meals or post glucose load [25]. Therefore, postchallenge hyperglycemia may trigger the interaction between oxidative stress, remnant-like lipoproteins, LDL-C, and sdLDL and subsequently elevate the risk of cardiovascular complications. Our study showed that postchallenge hyperglycemia at all the different time-course (30, 60, 90, and 120 min) and GluAUC was strongly related to arterial stiffness; therefore, it is reasonable to postulated that postchallenge hyperglycemia could have additive effects with atherogenic lipoprotein particles (LDL and sdLDL) on subclinical atherosclerosis, former research have indicated that glucose-mediated elastic artery sclerosis may play an integral role in the development of macrovascular complications [26]. The additive effects of atherogenic LDL-C (or sdLDL-C) and different glycemic indices on arterial stiffness demonstrated that postchallenge hyperglycemia have similar effects on subclinical atherogenesis compared with fasting glucose or HbA1C, particularly in subjects with higher sdLDL-C or LDL-C levels.

Additionally, our study also provides direct evidence that the occurrence of hyperglycemia, which was independent from the status of postchallenge, fasting, or HbA1C, may potentiate the effect of atherogenic lipoprotein particles (LDL and sdLDL) on the risk of subclinical atherosclerosis. The findings may also further support the results of elegant large-scale lipid-lowering trials among diabetes that demonstrate that with statin treatments can significantly slowing atherogenic progress. Thus, may contribute the reduction of cardiovascular morbidity and mortality risk [27].

Moreover, our results also are in agreement with those previous studies reported that an increase in sdLDL particles and increased postchallenge hyperglycemia may lead to an elevated risk of subclinical CVD [28, 29]. Also, based on LDL particle size has been found to be the strongest marker of clinically apparent and nonapparent atherosclerosis in patients with type 2 diabetes [30]. These studies further displayed that quantitative sdLDL-C measurements could provide alternative useful information for the risk assessment of atherosclerotic disease in addition to LDL-C [31].

It is well known that diabetes is a complicated disease that involves different levels of cellular dysfunction and multiple organs; hyperglycemia accelerated arterial stiffening by increasing the formation of AGE, which alters vessel wall structure and function. The irreversible formation and deposition of reactive AGE may most likely be the pathway involved in the pathogenesis of accelerated atherosclerosis in patients with diabetes [32]. The atherogenic effects of both fasting and postprandial hyperglycemia were ascribed to glucose-mediated cellular properties via several mechanisms such as an increase in nonenzymatic glycation of proteins and lipids. The findings of this study enhanced our previous work that postchallenge hyperglycemia is associated with the subclinical atherosclerotic marker cardio-ankle vascular index of arterial stiffness [24]. In another study involving 582 individuals aged 40–70 years and at risk for type 2 diabetes, postchallenge plasma glucose levels (30, 60, 90, and 120 min) as well as GluAUC were more strongly associated with carotid IMT compared with fasting glucose and HbA1c levels [28]. However, applying interaction analyses to test the concurrent atherogenic lipids and glycemic indices provides a deeper view of the interaction between lipids and hyperglycemia on the development of atherosclerosis.

Studies from other Asian populations have demonstrated that postchallenge glucose levels may be related to CVD events in patients without diabetes [33, 34]. Combining the additive effects atherogenesis observed during the hyperglycemic state of the present study also showed a higher prevalence of cardiovascular risk factors among patients belonging to the highest tertile. It is reasonable to assume that the postchallenge hyperglycemic spikes may account for the aforementioned increased CVD events. Therefore, postchallenge hyperglycemia should be emphasized as a target for decreasing the incidence of diabetes and CVD [35].

Recently, a research conducted in a type 2 diabetes and prediabetes population has successfully displayed the relationship between sdLDL with carotid IMT and insulin resistance [36]. In addition to the traditional risk markers, sdLDL has become an independent risk marker for adverse metabolic status such as dysglycemia, indicating that more attention should be paid to hyperglycemia which occurs independently from fasting, postchallenge, or postprandial in subjects with atherogenic dyslipidemia and particularly high levels of LDL-C or sdLDL-C to serve as a primary prevention of CVD.

The present study should be interpreted in consideration of some limitations. First, the study conducted within non-diabetes patients instead of diabetes patients. However, in order to eliminate confounding factors such as chronic diabetes-related complications and interventions, our study evaluated information gathered from adults without diabetes. Second, even though HbA1c was not statistically and significantly associated with baPWV in the presented study, based on this cross-sectional study, we cannot definitely make the conclusion that postprandial hyperglycemia are more important than HbA1C in the association with cardiovascular diseases. Last, the exact mechanisms of interactions between glucose and lipids profiles need to be elucidated in future study.

## Conclusions

In conclusion, significant interactions between LDL subclass particles and different glycemic indices displayed an additive effect between these two risk factors on arterial stiffness. In addition, only concurrent highest tertile levels of both glycemic indices and atherogenic LDL-C or sdLDL-C have significant risk for arterial stiffness in nondiabetic adults.

## Methods

From 2009 to 2011, we recruited 592 middle-aged (age range, 20–60 years old) adults without clinical diabetes (fasting plasma glucose <7.0 mmol/L or 2-h plasma glucose <11.1 mmol/L after OGTT) or known history of CHD or stroke to participate as the control group of “Work-related factors and cardiovascular events in patients with coronary heart disease” study conducted in the National Taiwan University Hospital, Taipei, Taiwan [37]. The present study of below diagnostic OGTT diabetes criteria was defined as 2-h glucose ≥11.1 mmol/L after OGTT but without previously known clinical diabetes as above mentioned. Informed consent was obtained prior to participation. The body mass index was calculated by the formula: weight (kg)/[height (m)]^2^. Smoking habit was defined as persons who had smoked more than 100 cigarettes in their lifetimes and still smoke at the time of the study. Hypertension is defined as SBP values >140 mmHg and/or DBP >90 mmHg, alcohol consumption. The alcohol consumption was defined as up to 1 drink per day for women and up to 2 drinks per day for men. This study was approved by the ethics committee of National Taiwan University Hospital.

### Blood sampling for lipids and biochemical studies

Following overnight fasting for 10–14 h, the subjects underwent a standard 75-g oral OGTT to evaluate their response to glucose loading. A venous blood sample was obtained through the antecubital vein before OGTT and at 30, 60, 90, and 120 min after OGTT. The glucose area under curve (GluAUC) for each subject was calculated by the five times (0, 30, 60, 90, and 120) of the blood glucose level sample after OGTT. The postprandial period was defined to extend until 120 min after meal onset, baseline (0) was defined as the pre-meal plasma value. The assessment of levels of lipids, including cholesterol, triglycerides, HDL-C, and LDL-C, Cholesterol within small dense LDL (15.0 nm-20.0 nm) was measured as described previously [38, 39] using a newly developed automated homogeneous assay (Denka Seiken Co., Ltd., 3-4-2 Nihonbashi-Kayabacho, Chuo-Ku, Tokyo) and analyzed on an autoanalyzer (Toshiba FR-200 automatic chemistry analyzer, Tokyo, Japan). Subject’s blood samples were collected in the morning under 10–14 h fasting conditions, after which the blood sample were centrifuged for 10 min at 3,000 revolutions per minute. The serum was separated and stored at -80°C. The blood collection to separation process did not exceed 3 hours under proper laboratory protocol.

### Brachial-ankle pulse wave velocity measurements

The brachial-ankle pulse wave velocity (baPWV) was measured in the morning after participants received OGTT using a noninvasive vascular screening device (Colin VP-1000; Colin Co., Ltd., Komaki, Japan). This device simultaneously recorded the baPWV, electrocardiogram, and arterial blood pressure from all four limbs (at both the left and right brachia and ankles) and calculated the ankle-brachial index. Subjects were examined in the supine position. Electrodes were placed on both wrists to obtain an electrocardiogram. The cuffs were connected to a sensor that measured the volume pulse form and an oscillometric pressure sensor that measured blood pressure. The time interval between the wave front of the brachial waveform and the ankle waveform was defined as the pulse transit time between the brachia and the ankle (ΔTba). The distance between the arm and ankle was estimated automatically according to the subject’s height. The distance from the heart to the brachia (Db) was measured using the following equation: Db = 0.2195 × height of the patient (cm) -2.0734. The distance from the heart to the ankle (Da) was measured using the following equation: Da = 0.8129 × height of the patient (cm) +12.328. The baPWV was calculated using the following equation: baPWV = (Da - Db)/ΔTba. The validity and reproducibility of baPWV measurements has been reported elsewhere [20, 21].

### Statistical analysis

Data analysis was performed using Statistical Analysis System (SAS) software (version 9.2, SAS Institute Inc, Cary NC, USA). The statistical significance level was set at 0.05, and the power calculation was set with a 1-beta value of 80% for this study. Continuous variables were expressed as means ± standard deviations and categorical data was expressed by percentage. The statistical methods used to test the significance of the compare continuous variables (Tables 1 and 2) were performed with CA (Cochran-Armitage) test for trend.

Basic information of cardiovascular characteristics and subclinical atherosclerotic markers was compared by Chi-square test for trend across the tertile distribution of the groups using glucose levels during postchallenge hyperglycemic spikes 1 h after the OGTT, which was performed to demonstrate postchallenge hyperglycemic status. The trend differences between the cardiovascular characteristics of the patients across the tertile distributions of the groups for baPWV were also evaluated.

The effects of LDL-C (or sdLDL-C) and glucose levels at the different time points of OGTT results were measured using multiple logistic regression models to estimate the odds ratios and 95% confidence intervals for arterial stiffness in terms of baPWV at the level of ≥75th percentile. This analysis was performed after controlling for related covariates, including age, gender, BMI, hypertension, smoking, and alcohol consumption. Since there were high correlations observed between the different glycemic indices and atherogenic LDL-C (or sdLDL-C), the concurrent presence of these two components was treated as interaction to test their risk for arterial stiffness.

## Abbreviations

CVD: Cardiovascular disease
CHD: Coronary heart disease
LDL: Low-density lipoprotein
LDL-C: Low-density lipoprotein cholesterol
FPG: Fasting plasma glucose
OGTT: Oral glucose tolerance test
HbA1c: Glycated hemoglobin
GluAUC: Glucose area under curve
HDL-C: High-density lipoprotein cholesterol
sdLDL-C: Small dense LDL-cholesterol
IMT: Intima-media thickness
baPWV: Brachial-ankle pulse wave velocity
eGFR: Estimated glomerular filtration rate.
